# Comparative proteome analysis of the tegument of male and female adult *Schistosoma mansoni*

**DOI:** 10.1038/s41598-022-11645-3

**Published:** 2022-05-09

**Authors:** Franziska Winkelmann, Manuela Gesell Salazar, Christian Hentschker, Stephan Michalik, Tomáš Macháček, Christian Scharf, Emil C. Reisinger, Uwe Völker, Martina Sombetzki

**Affiliations:** 1 Division of Tropical Medicine and Infectious Diseases, Center of Internal Medicine II, Rostock University Medical Center, Rostock, Germany; 2 Department of Functional Genomics, Interfaculty Institute for Genetics and Functional Genomics, University Medicine Greifswald, Greifswald, Germany; 3 Department of Parasitology, Faculty of Science, Charles University, Prague, Czech Republic; 4 Department of Otorhinolaryngology, Head and Neck Surgery, University Medicine Greifswald, Greifswald, Germany

**Keywords:** Biological techniques, Diseases, Pathogenesis

## Abstract

The tegument, as the surface layer of adult male and female *Schistosoma* spp. represents the protective barrier of the worms to the hostile environment of the host bloodstream. Here we present the first comparative analysis of sex-specific tegument proteins of paired or virgin *Schistosoma mansoni*. We applied a new and highly sensitive workflow, allowing detection of even low abundance proteins. Therefore, a streptavidin–biotin affinity purification technique in combination with single pot solid-phase enhanced sample preparation was established for subsequent LC–MS/MS analysis. We were able to identify 1519 tegument proteins for male and female virgin and paired worms and categorized them by sex. Bioinformatic analysis revealed an involvement of female-specific tegument proteins in signaling pathways of cellular processes and antioxidant mechanisms. Male-specific proteins were found to be enriched in processes linked to phosphorylation and signal transduction. This suggests a task sharing between the sexes that might be necessary for survival in the host. Our datasets provide a basis for further studies to understand and ultimately decipher the strategies of the two worm sexes to evade the immune system.

## Introduction

Schistosomiasis affects about 240 million people worldwide, causing approximately 200,000 deaths annually^1^. Infection leads to chronic disease causing a global burden of 1.9 million disability-adjusted life years (DALYs)^2^. The causative pathogens are parasitic blood flukes (trematode worms) of the genus *Schistosoma* spp. They survive for decades in their mammalian final hosts, including humans, without being attacked by the immune system. The anthelmintic drug Praziquantel, used for treatment of schistosomiasis, only affects adult stages in the final host and does not prevent recurrent infections^3^. Thus, a vaccination against schistosomiasis is ranked among the 10 most urgently needed vaccines^4^.

Gonochorism and phenotypic sexual dimorphism, displayed by intramammalian schistosome stages, is quite unique among trematodes^5,6^. The surface of adult worms, the tegument, is a syncytial cytoplasmic layer that represents the interface between parasite and host. It forms a protective barrier that shields the parasites not only from the host immune system but also from physical influences to which they are exposed in the host bloodstream. It is internally terminated by a basement membrane and externally by a multilaminar membrane consisting of an inner plasma membrane with an overlying membranocalyx. The cytoplasm is connected to the cell body by microtubule-stabilized bridges located beneath the peripheral muscle layer^7^. Of note, the tegument also exhibits sex-specific morphological and biochemical differences. The dorsal surface of male worms is characterized by large tubercles covered laterally with rigid intracellular spines, while the surface of the gynecophoric canal is ribbed and covered with small, irregularly arranged spines. In contrast, female worms possess only a few spines, most frequently found at their posterior end, and their surface is smoother, without tubercles, but in contrast to the tegument structure of male worms, they show a pronounced enlargement^8–11^. Proteomic analyses of adult schistosome teguments have been performed using various extraction techniques^12–19^. There are few studies indicating sex-specific differences in the tegument proteome of adult paired *Schistosoma japonicum*^16,20,21^. Such differences can be associated with sex-specific effects of schistosome infection on the host^22–24^.

Further, it has been shown that the developmental stage of male and female adult worms depends on mating status^25,26^. A total of 318 transcripts, 265 in females and 53 in males, are differentially expressed in paired and virgin (from unisexual infection) worms^27^. These differences are reflected in behavioral, physiological and antigenic differences between virgin and paired worms. Males from unisexual infections show lower body weight and are more active^28–31^. Females from unisexual infections are developmental stunted and show a premature reproductive system^30,32,33^.

Characterizing the molecular host-parasite interface is critical for a better understanding of the sex-specific biology of schistosomes and the development of new drugs for infection control. We therefore investigated the tegument of male and female schistosomes, including worms from unisexual infection. Using a single pot solid-phase enhanced sample preparation (SP3) protocol we were able to detect a large number of proteins with low abundance and from small amounts of material, such as the tegument of adult *S. mansoni*.

## Results

### Specific biotin tagging of the outer surface of adult *Schistosoma mansoni*

**Figure 1:**
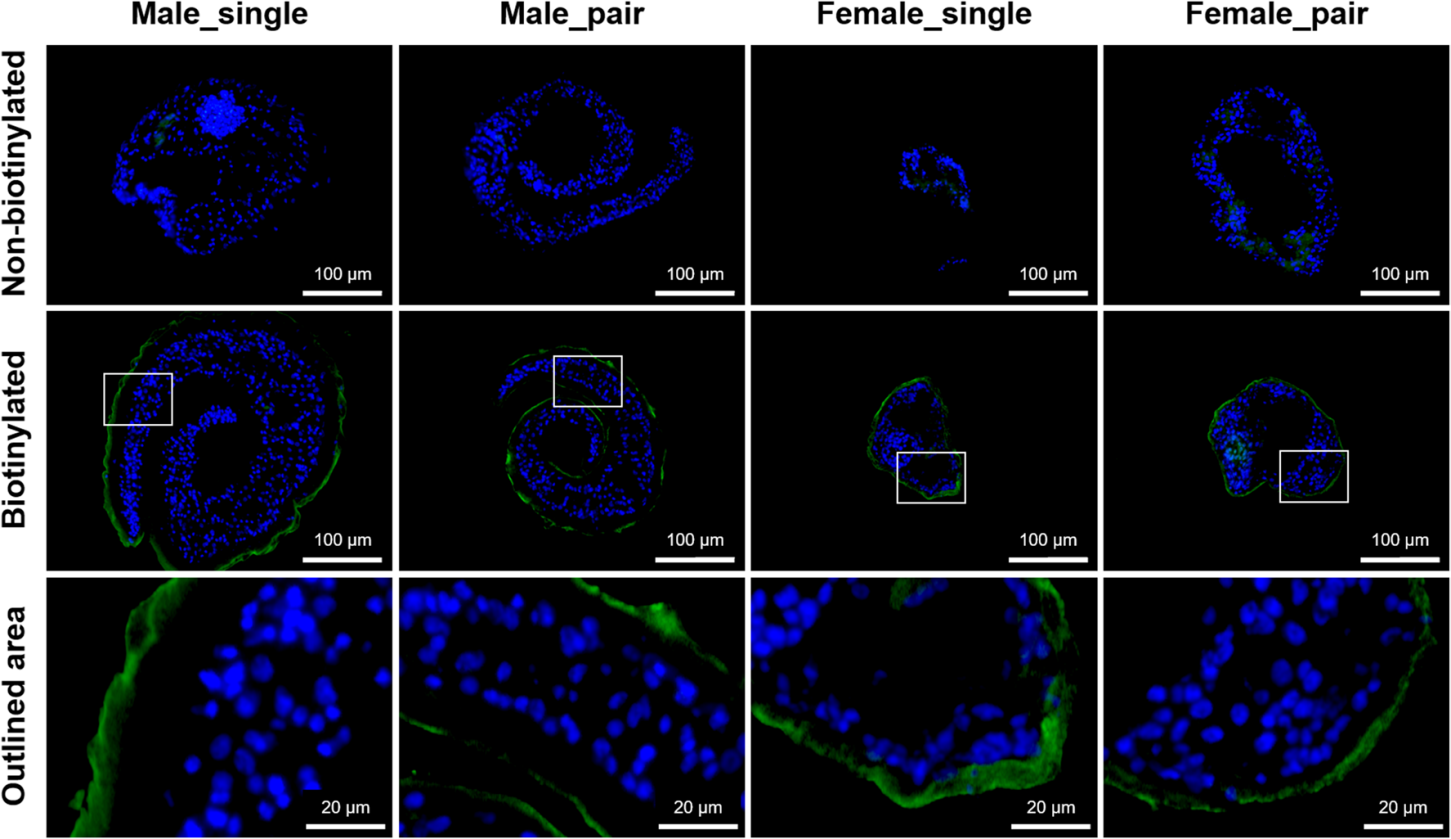
Specifically bound biotin tags the outer surface of adult *Schistosoma mansoni*. Images obtained by fluorescence microscopy showing representative surface biotinylation of male_single, male_pair, female_single and female_pair adult worms. The worms were incubated with biotin and cross cryosections were then prepared and probed with avidin-FITC (green). Nuclei were stained with DAPI (blue). The specific avidin-FITC signal was detected only in the biotinylated surface tegument in all groups. The difference in size of biotinylated male_single (compared to other male groups) is only due to a different position at which the cryosection was performed.

To ensure that tegument proteins at host-parasite interface were preferentially isolated, *S. mansoni* paired and virgin adult worms were biotinylated using EZ-Link Sulfo-NHS-SS-Biotin and examined by fluorescence microscopy to verify the biotin labelling efficiency and biotin distribution pattern. The images in Fig. 1 show adult worm cross sections of male_single, male_pair, female_single and female_pair after avidin-FITC incubation and DAPI staining. A clear and intense green signal was detected over the entire surface tegument. However, no signal was observed in the internal organs and gastrodermis of the adult worms, indicating selective labelling of tegument proteins. Control sections of non-biotinylated adult worms show no fluorescence and only the staining of the nuclei with DAPI was observed.

### Sample preparation via SP3 protocol ensures high identification rates of proteins from a small amount of tegument material from adult *Schistosoma mansoni*

**Figure 2:**
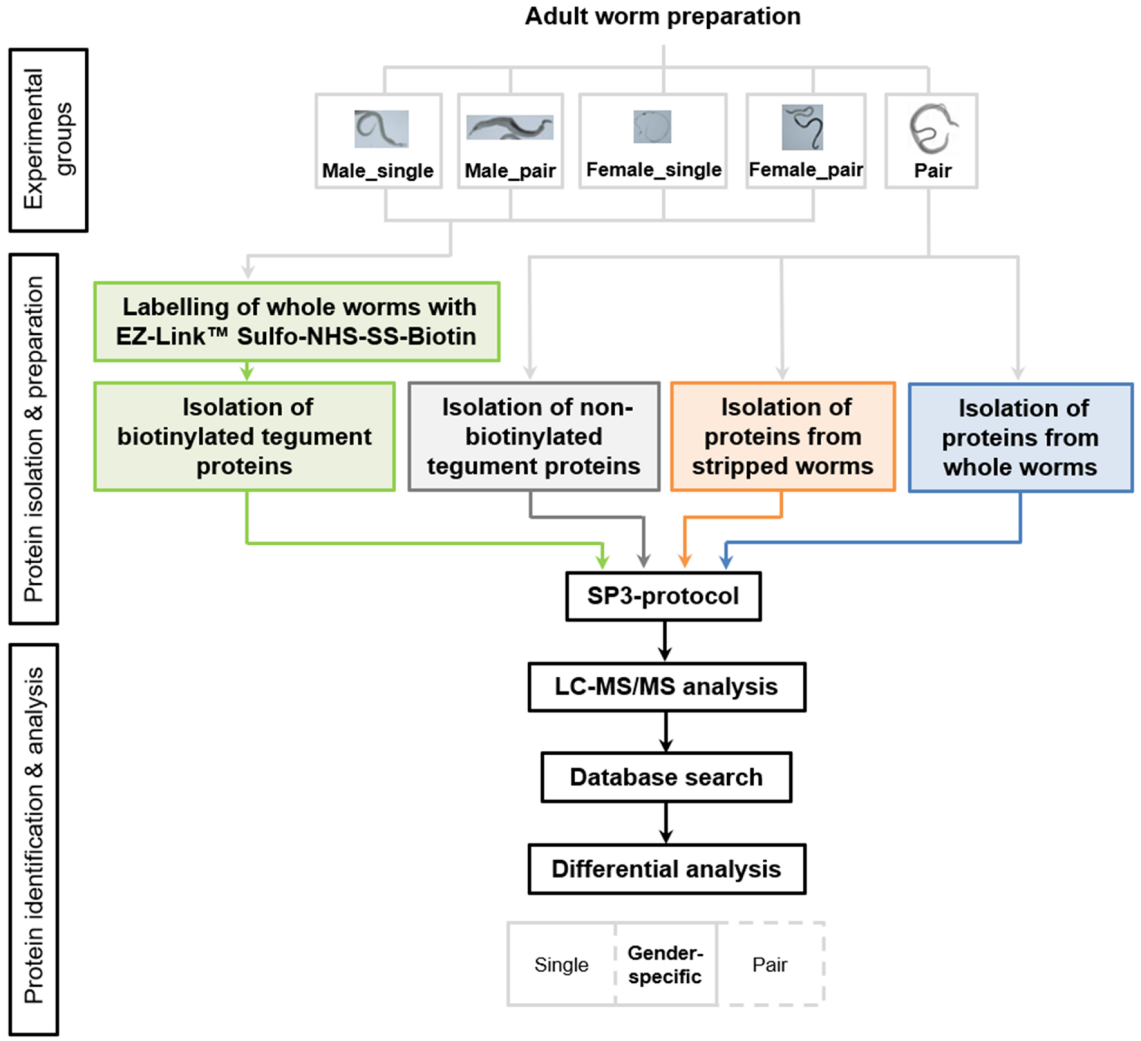
Research workflow showing the identification process of proteins of adult *Schistosoma mansoni*. Surfaces of paired and virgin adult schistosomes were biotinylated to extract surface proteins at host-parasite interface. Tegument samples were processed with the Pierce Cell Surface Protein Isolation Kit to isolate biotinylated proteins. Proteins of whole and stripped worms were obtained by their homogenization. Protein extracts were prepared for following LC–MS/MS via single pot solid-phase enhanced sample preparation protocol and protein candidates were identified over database search followed by differential analysis. Thus, gender-specific proteins of adult worms are presented in this study regardless of their stage of development.

We applied a new workflow to identify tegument proteins of adult *S. mansoni* (Fig. 2). Specifically, we used a single pot solid-phase enhanced sample preparation (SP3) protocol to improve the identification of proteins from small amounts of starting material, such as the tegument of adult *S. mansoni*. In order to test the efficiency of our protocol and to focus our analyses on the tegument proteins, we have included a number of controls. First, we analyzed protein extracts from whole worms (without biotinylation). Here, we identified 1112 proteins and 8684 peptides from 25 worm pairs separated by hand (50 worms: 25 females and 25 males). Similarly, we have removed the tegument of worms and analyzed these so-called “stripped” worms (without tegument, without biotinylation) from 175 worm pairs. In this control group we were able to detect 1189 proteins (9465 peptides) (Supplementary Table S1).

**Figure 3:**
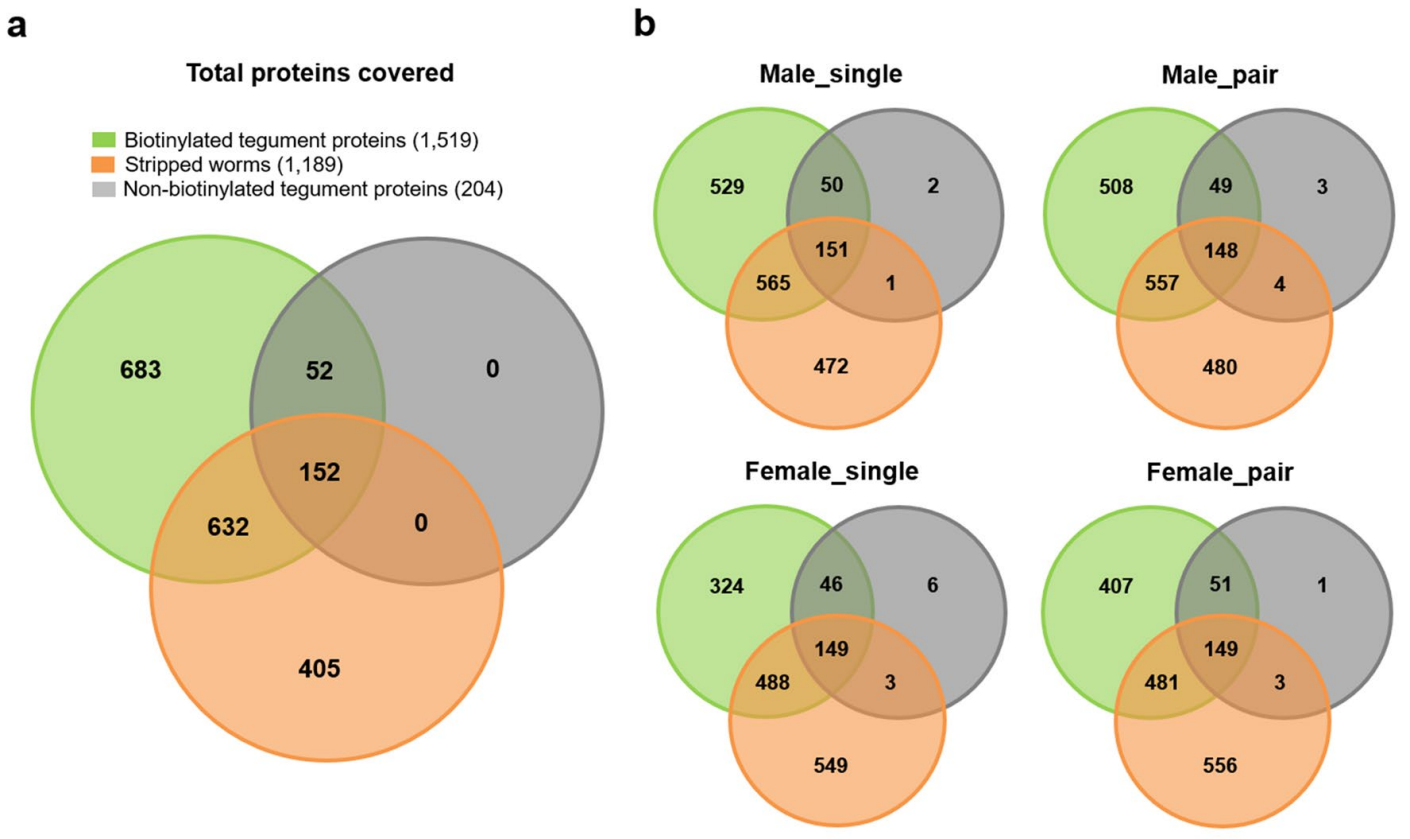
Identified proteins in adult *Schistosoma mansoni* proteomes. Venn diagrams representing the total amount (**a**) and the group-specific amount (**b**) of biotinylated tegument proteins (green), non-biotinylated tegument proteins (grey) and proteins of stripped worms (orange) in different adult *Schistosoma mansoni* worm proteomes.

In our experimental groups (male_pair, male_single, female_pair, female_single) consisting of tegument protein fractions isolated by enrichment of biotinylated proteins we were able to detect 1519 proteins (9668 peptides) and 204 proteins (1013 peptides) in the non-biotinylated tegument control (Supplementary Table S1). To exclude biotin-containing proteins or non-specific binding we removed the non-biotinylated tegument control as well as the proteins of the stripped worms from our dataset (Fig. 3a). Of note, more than half of the total biotinylated proteins were also found in the control group of stripped worms (Fig. 3a). We identified more proteins in male than in female worms (biotinylated without controls: male_pair 508; male_single 529; female_pair 407 and female_single 324), regardless of whether paired or virgin. This also applies to the non-biotinylated controls and the stripped worms (stripped without biotinylated: male_pair 480; male_single 472; female_pair 556 and female_single 549) (Fig. 3b). Interestingly, the highest number of non-biotinylated tegument proteins (not present in biotinylated tegument proteins or stripped worm) was detected in the female_single group, with a number of six proteins versus two (male_single), three (male_pair), and one (female_pair). These proteins could be assumed as natively biotin-carrying proteins. Furthermore, biotinylated tegument protein fractions of male worms share a higher proportion of proteins with stripped worms in comparison to the female tegument fraction (shared biotinylated and stripped: male_pair 557; male_single 565; female_pair 481 and female_single 488) (Fig. 3b).

### Male- and female-specific proteins of adult *Schistosoma mansoni*

**Figure 4:**
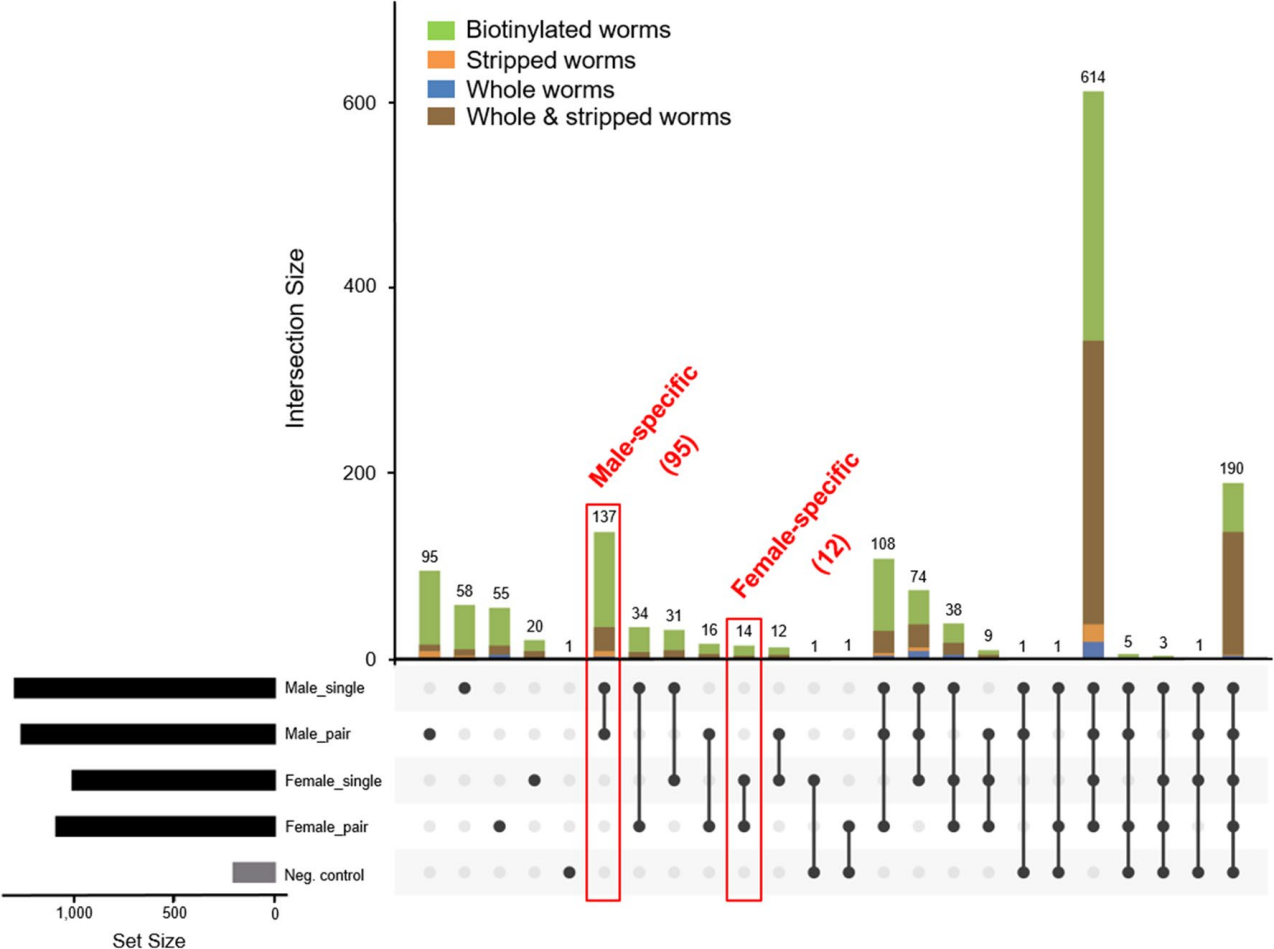
Identified male- and female-specific proteins of adult *Schistosoma mansoni*. The numbers and intersections of the identified proteins from different *Schistosoma mansoni* proteomes was visualized using an Upset plot. Only proteins from *S. mansoni* were included. Connected dots display shared proteins between datasets, and the total number of proteins identified in a particular dataset is indicated in the set size. Number of male- and female-specific proteins after subtraction of the control groups non-biotinylated tegument proteins (= neg. control) and stripped worm proteins, shown in red.

Further, we screened for proteins that were present in both, male_single and male_pair or female_single and female_pair. These proteins were referred as male-specific and female-specific, respectively (highlighted in Fig. 4, Supplementary Table S2). Following subtraction of the proteins from the control groups, the negative control (non-biotinylated tegument proteins) and proteins from stripped worms, 95 male-specific and 12 female-specific proteins were subjected to further analysis. In addition, we were able to detect protein fractions that were found exclusively in the respective experimental groups: male_pair, 72; male_single, 45; female_pair, 38 and female_single, 11 (Fig. 4, Supplementary Table S1).

Among the male-specific proteins (Supplementary Table S2), one saposin-containing protein (G4LVS4), a glycerol-3-phosphate dehydrogenase (G4VNX4), and one uncharacterized protein (G4V8L1) contained a signal peptide (3.2%). Another ten proteins (9.5%, like putative calponin homolog, G4LX37, or putative lipopolysaccharide kinase, G4LYN6) displayed transmembrane domains. Of these few remaining female-specific proteins (Supplementary Table S2), one saposin B-type domain-containing protein (G4VHH4) comprised a signal peptide, and five proteins (41.7%, such as inositol transporter, G4VE30, or putative cytochrome B5, G4VP31) had transmembrane domains.

### Subcellular localization of biotinylated tegument proteins from male and female *Schistosoma mansoni*

Male- and female-specific tegument proteins were classified into six groups according to their predicted subcellular location using CELLO2GO (Supplementary Fig. S1). Overall, 25 male-specific proteins (26.3%) and four female-specific proteins (33.3%) were assigned to the plasma membrane. Likewise, 26 male-specific proteins (27.4%) and four female-specific proteins (33.3%) were predicted to be cytoplasmic. Additionally, 74 male-specific proteins (77.9%) and only five female-specific proteins (41.7%) were assigned to the nucleus. Exclusively, male-specific group contained four (4.2%) extracellular proteins and one (1.1%) endosomal protein. Furthermore, the male-specific group contains five (5.3%) and female-specific group four (33.3%) mitochondrial proteins.

### Gene ontology analysis of biotinylated tegument proteins from male and female *Schistosoma mansoni*

The tegumental proteins of male- and female-specific group were subjected to a GO-enrichment analysis using g:Profiler (Supplementary Fig. S2, Supplementary Table S2). For male-specific proteins there was an enrichment apparent for those proteins assigned to metabolic processes, protein modification process like phosphorylation, signal transduction and also transport processes for example via vesicles (Supplementary Fig. S2). Due to the small size of the female-specific group no enriched categories could be identified.

### Protein family (Pfam) analysis of biotinylated tegument proteins from male and female *Schistosoma mansoni*

Gender-specific proteins of adult worms were also subjected to a Pfam analysis. The 10 most represented protein domains for male-specific and female-specific adult *S. mansoni* are shown in Supplementary Fig. S3 and Supplementary Table S2. While in the female-specific group there is no accumulation of a specific domain, in the male-specific group there is an enrichment of Protein kinase domain and Phorbol esters/diacylglycerol binding domain (C1 domain). Interestingly, among the 12 female-specific proteins, no protein domain was found that belongs to the 10 representative protein domains for male-specific group.

## Discussion

This study was conducted to identify sex-specific tegument proteins from adult *S.* *mansoni* to provide evidence for their immune evasion strategies. Using a new, improved workflow, including the SP3 protocol, we were able to considerably increase the identification rates of proteins from the tegument of adult *S. mansoni*. In total, 1519 proteins were identified in the biotinylated protein samples of all experimental groups: male_single, male_pair, female_single and female_pair.

In a previous study by Zhang et al*.*, 479 proteins were identified as tegument exposed in separated adults of *S. japonicum* using similar techniques^16^. In 2013, in vivo intravascular biotinylation of *S. bovis* adult worms was used for proteomic analysis of tegumental surface proteins with the result of 108 identified proteins^17^. Of note, 662 tegument proteins from adult *S. haematobium* were found by combining a comprehensive protein fractionation approach consisting of OFFGEL electrophoresis with high-throughput mass spectrometry^19^. In our study, 784 of the 1519 identified biotinylated proteins were also found in stripped worms, which may indicate incomplete detachment of the tegument during protein extraction. Other authors have attributed the presence of tegument proteins in stripped worms to their expression in the cell bodies below the tegument syncytium, which are thus detectable there as well^14^. Compared to a previous study by van Balkom et al*.*, we identified significantly more proteins for stripped worms^12^. Taken together, we demonstrated that our approach based on the SP3 protocol markedly extended the available data in very small raw material. Thus, we provide a deeper and more comprehensive insight into the *S. mansoni* tegument proteome.

We demonstrated that male schistosomes, whether derived from bi- or unisexual infection, have more specific tegument proteins than females. The higher biomass of male worms might explain the increased identification rate. In contrast, virgin females are significantly smaller compared to paired females, which in turn is reflected by significantly less identified proteins in the group female_single compared to female_pair^29^. In line, Cheng et al*.* and Zhang et al*.* showed that female worms express relatively low numbers of female-specific proteins^12,16,21^. Our data confirm these findings with 95 male-specific vs. 12 female-specific proteins out of a total of 683 identified proteins, after subtraction of the controls (Fig. 4, Supplementary Table S2). Zhang et al*.*, Cheng et al*.* and Liu et al*.* also identified sex-specific tegument proteins. However, they have focused on paired male and female worms^16,20,21^. In contrast, we included virgin adult worms derived from unisexual infections to increase the sex- specificity by reducing the impact of mating. Worm pairing initiates extensive changes in female worms, such as maturation and oviposition. Such morphological changes are not visible in male worms. On transcriptomic level differences between paired and virgin schistosomes have already been shown^27^. The sexual development, so-called "magic" between the worm sexes, is not yet fully understood^34,35^. However, we showed that in both groups, male_paired and female_paired, the number of detected proteins was higher compared to male_single and female_single, respectively. We refer to this here as the "mating influence" on the tegument, which was excluded by matching the datasets of paired and virgin worms.

Next, we focused on the function of individual sex-specific proteins. Of them, a myo-inositol transporter (Smp_134080; G4VE30) was found exclusively in female worms (Supplementary Table S2). Related proteins have already been described and characterized in other parasites. In *Trypanosoma brucei*, a parasite that causes African sleeping sickness, an H^+^/inositol transporter, TbHMIT, was identified. It has been proven to be essential for the survival of the pathogen in culture^36^. Furthermore, H^+^/myo-inositol transporter genes, *hmit-1.1* and *hmit-1.2*, have roles in the osmo-protective response in *Caenorhabditis elegans*^37^. Myo-inositol is an important component of metabolic pathways and forms the structural basis of phosphatidylinositol, a membrane compound that in turn serves as an educt for the synthesis of phosphorylated derivatives, the phosphoinositides^38^. Interestingly, genes involved in the phosphatidylinositol signaling pathway may play important roles in a variety of cellular processes, including membrane transport, cell motility, cytoskeletal reorganization, DNA synthesis, the cell cycle, adhesion, signal transduction and reproductive systems of *S. mekongi*^39^. However, nothing is known about inositol biosynthesis in schistosomes so far.

A significant enrichment of the Pfam domains Protein kinase domain and Phorbol ester/diacylglycerol binding domain (C1 domain) was found for male-specific proteins (Supplementary Fig. S3). This is accompanied by GO-enrichment for those genes that are assigned to protein modification processes, more specifically protein phosphorylation and genes involved in signal transduction (Supplementary Fig. S2). Protein kinases catalyze the apposition of phosphate groups to substrate proteins in order to alter the properties of the target proteins such as enzyme activity, localization, conformation, interactions with other proteins, or to mark them for destruction. The kinome of *S. mansoni* comprises 268 protein kinases (~ 1.9% of the total proteome), similar to those reported for *S. haematobium*^40,41^. Protein kinase B (PKB) has been described to regulate glucose uptake in *S. mansoni* from host blood to support growth, development and reproduction through cellular signal transduction^42^. Furthermore, protein kinases C (PKCs) and extracellular signal-regulated kinases (ERKs) of *S. mansoni* appear to play a central role in signaling pathways associated with movement, attachment, mating and egg release^43^. In addition, the mitogen-activated protein kinase (MAPK) Smp38 has been proposed to regulate essential signaling pathways for survival, oviposition and antioxidant defense of adult *S. mansoni*^44^. Hirst et al*.* have shown that female worms display a higher protein phosphorylation rate compared to male worms^45^. In contrast, we found an enrichment for protein kinases exclusively among male-specific tegument proteins. The roles of protein kinases in adult worm signaling pathways appear to be miscellaneous and important for the maintenance of adult worm homeostasis. However, protein phosphorylation in schistosomes, or even specifically in the tegument, is poorly understood. The male-specific protein kinases highlighted here could provide evidence to specific functions of adult male *S. mansoni*.

Nascent proteins are thought to be directed into their final subcellular compartments already during translation, based on specific parts of their amino acid sequence. However, prediction of subcellular localization is difficult for schistosomal proteins due to the low homology of the proteins outside the genus, which complicates functional annotation^46^. To date, many prediction methods of the subcellular localization of eukaryotic proteins are mainly based on the targeting of signal, sequence-based, or annotation-based features^47^. Identified tegument proteins from different proteomic analyses have shown high diversity of predicted subcellular locations^15–18^. In our study, 25 male-specific proteins (26.3%) and only four female-specific proteins (33.3%) are predicted to be localized in the plasma membrane, while other predicted membrane-associated proteins could be localized in the nucleus, mitochondrion and other cell organelles (Supplementary Fig. S2). Tetraspanins (TSP), a family of integral membrane proteins, are ubiquitously found on the plasma membranes of eukaryotic organisms. Significantly, in schistosomes, they represent potential candidates for the development of new diagnostic assays, and they are discussed as potential vaccine candidates^48–50^. They have been shown to be necessary in schistosomes for tegument formation, maturation, and stability^51^. TSPs have been detected in different life stages of schistosomes^51–53^. Here, we also detected 12 TSPs in male and female adult worms, including Sm-TSP-1 (Q86D97) and Sm23 (G4LW70). Compared to other studies that focused on the tegument proteome of adult worms, we did not detect Sm-TSP-2. So far, however, it appears that the prediction of subcellular locations indicative of functions in host-parasite interactions is limited for schistosomes, so that final localization needs to be verified by further methods, such as immunolocalization.

To our knowledge, this is the first time that comparative tegument proteomic analyses of adult schistosomes have been performed, examining male and female and paired and virgin worms concurrently. We can summarize that male-specific proteins were found to be enriched in processes linked to phosphorylation and signal transduction, whereas female-specific proteins are rather associated with signaling pathways of cellular processes and antioxidant mechanisms. This suggests a task sharing between the sexes that might be necessary for survival in the host. Our datasets provide a basis for further studies to understand and ultimately decipher the strategies of the two worm sexes to evade the immune system.

## Methods

### Ethics statement

All animal experiments were conducted in strict compliance with the regulations of the German Society for Laboratory Animal Science, the European health guidelines of the Federation of Laboratory Animal Science Associations and in accordance with ARRIVE guidelines 2.0. The protocol was approved by the local Animal Research Committee (Landesamt für Landwirtschaft, Lebensmittelsicherheit und Fischerei (LALLF) of the state Mecklenburg-Westernpommerania (LALLF M-V/TSD/7221.3-2-022/17). All efforts were made to minimize animal suffering.

### *Schistosoma mansoni* life cycle and isolation of adult worm

*Schistosoma mansoni* (Belo Horizonte strain) was kept in a life cycle using *Biomphalaria glabrata* fresh water snails as intermediate hosts and 6–8 weeks old female NMRI mice as definitive hosts, as previously described^54^. To obtain either male or female cercariae for subsequent infection of mice, individual *B. glabrata* were exposed to a single *S. mansoni* miracidia. Single-sex cercariae were harvested six weeks later. The sex of the cercariae was determined by DNA amplification of sex-related chromosome segments using female-specific primers as previously described^23,55^. The mice were percutaneously infected with 300 *S. mansoni* cercariae, either male, female, or mixed in equal proportions. On day 49 post infection mice were sacrificed via cervical dislocation under ketamine/xylazine anesthesia. Adult worms were collected by perfusion of the portal venous system as previously described^56^. Worms were washed three times with washing buffer (RPMI with 100 U/ml penicillin and 100 µg/ml streptomycin). Following microscopic evaluation, intact adult worms were incubated in culture medium [RPMI with 100 U/ml penicillin, 100 µg/ml streptomycin and 10% inactivated fetal bovine serum (Thermo Fisher Scientific, Germany)] at 37 °C in a humid atmosphere containing 5% CO_2_.

### Workflow for the identification of proteins of adult *Schistosoma mansoni*

The experimental design includes the following experimental groups: male and female worms out of a pair (male_pair; female_pair) and male and female worms originated from single-sex infection (male_single and female_single). About 350 worms were used for each group. As a control group, a sample of 50 worms (from pairs) was also prepared for the measurement of the total worm proteome. Another 350 adult worms were applied to analyze the tegument proteome without biotinylation as well as stripped worms (without tegument). Apart of the control groups, two independent infections and test runs were carried out as biological duplicates for the experimental groups (see Fig. 2).

### Preparation of biotinylated tegument proteins and proteins of whole and stripped adult worms

Surface proteins of vital adult worms were labeled with biotin as described previously^16^. In brief, intact adult worms were washed three times in ice-cold PBS (pH 7.4) and incubated in 10 ml of ice-cold PBS containing 0.89 mM membrane-impermeable sulfosuccinimidyl-2-(biotinamido)ethyl-1,3-dithiopropionate (EZ-Link Sulfo-NHS-SS-Biotin; Pierce, USA) for 30 min at 4 °C with gentle rotation mixing with subsequent quenching (1 ml of quenching solution; Pierce, USA). Worms were washed three times in ice-cold Tris buffered saline (TBS, pH 7.4) with protease inhibitors (protease inhibitor cocktail; Fermentas Life Sciences, USA) and snap frozen in liquid nitrogen. The success of the biotin labeling was checked under an immunofluorescence microscope (see below). The tegument of biotinylated and, as control, of non-biotinylated adult worms was stripped using a freeze–thaw–vortex method described by Roberts et al*.*^57^. Frozen worms were slowly thawed on ice and vortexed for 10 bursts of 1 s at maximum speed. Supernatants were collected and tegument material was pelleted by centrifugation at 1000×*g* for 30 min at 4 °C. We isolated the tegument proteins that we previously labelled (biotinylated tegument proteins) or that naturally exhibit a biotin labelling (non-biotinylated tegument proteins) using the Pierce Cell Surface Protein Isolation Kit according to the manufacturer's instructions (Pierce, USA; Fig. 2). In this procedure, the biotinylated proteins are captured with NeutrAvidin agarose and undergo affinity purification. Samples were eluted with 50 mM DTT in 0.1 M Tris–HCl (pH 6.8) to reduce disulphide bonds in the biotin label, resulting in the release of bound proteins without the biotin label, and mixed with SDS (5%). For the preparation of the remaining control groups, whole worms and stripped worms were homogenized in 0.1 M Tris–HCl (pH 6.8) using FastPrep-24 (MP Biomedicals, Thermo Fisher Scientific, Germany) at 2 × 6.0 m/s for 40 s. Homogenates were centrifuged at full speed for 3 min, supernatant was mixed with SDS (5%) and stored at − 80 °C.

### Sample preparation for proteomics

Samples from two independent infections were subjected to SP3 protocol, in which the whole samples with the appropriate worm numbers were processed^58^. The disrupted *Schistosoma mansoni* protein samples from the tegument fraction and the whole worm samples were reduced with 1 µl DTT (25 mmol, 30 min, 37 °C) and alkylated with 2 µl IAA (100 mmol, 15 min, 37 °C). Afterwards, the samples were prepared according to Blankenburg et al*.* for MS analysis^58^. In brief, 10 µl of prepared magnetic bead solution and acetonitrile were added to the samples for binding of the proteins. After washing with ethanol and acetonitrile, trypsin (100 ng) was added to the samples for overnight digestion. For elution of the peptides, 7 µl of 2% DMSO in H_2_O was added to the reaction pot. At the end, the supernatant without the beads and with the digested peptides was transferred into a vial with 7 µl prepared buffer (4% ACN and 0.2% acetic acid in H_2_O) for MS analysis.

### LC–MS/MS analysis

LC–MS/MS measurement was carried out using an Q Exactive plus mass spectrometer (Thermo Fisher Scientific) coupled to an Ultimate 3000 (Thermo Fisher Scientific) with a C18 Acclaim PepMap100 pre-column (inner diameter 100 μm, particle size 5 μm, pore size 100 Å, Thermo Fisher Scientific) and an analytical Accucore column (25 cm, inner diameter 75 μm, particle size 2.6 μm, pore size 150 Å, Thermo Fisher Scientific). Peptides were separated with a 120 min gradient of buffer A (aqueous solution of 0.1% acetic acid) and buffer B (0.1% acetic acid in acetonitrile) with a flow rate of 0.3 μl/min. Measurement was performed in DDA mode with top ten most abundant precursors being fragmented with HCD. Survey scan resolution was set to 70,000 with an AGC target of 3e6 and MS2 resolution was set to 17,500 with an AGC target of 2e5. The dynamic exclusion time was set to 30 s and the scan range from 300 to 1650 *m/z*. Mass spectrometry raw data from all measured samples and MaxQuant search results have been deposited to the ProteomeXchange Consortium via the PRIDE^59^ partner repository with the data set identifier PXD031167.

### Protein identification and bioinformatic analysis

MS/MS data were analyzed with MaxQuant (v.1.6.2.10) searching against a UniProt database limited to entries of *Schistosoma mansoni* and *Mus musculus* (v2018-05)*.* MS/MS search tolerance was set to 20 ppm without run alignment option and for digestion trypsin was specified with up to two missed cleavages. As fixed modification carbamidomethylation and as variable modification oxidation of methionine were considered. Peptide and protein FDR was set to 0.01.

MaxQuant search results were analyzed using R (version 4.0.1)^60^ with the tidyverse (version 1.3.0)^61^ & UpSetR package (version 1.4.0)^62^. The protein groups search results were filtered by removing contaminants and reverse protein groups hits. In addition, the dataset was filtered for protein groups found with at least 2 unique peptides in each of the two biological replicates per condition. The protein group identifications were compared between conditions using an UpSet plot^63^. Proteins were assigned to predicted function and subcellular location using CELLO2GO^64^. Putative transmembrane domain(s) and signal peptides were predicted using the program Phobius^65^. g:Profiler was used to classify proteins according to Gene Ontology (GO) categories, and protein family (Pfam) analysis was performed using Pfam 33.1^66,67^.

### Fluorescence staining of biotinylated tegument proteins

To validate the localization of biotinylated proteins, biotinylated and non-biotinylated (control) worms were embedded and frozen in OCT compound (Tissue-Tek, Sakura). Ten µm cryosections were prepared, transferred onto positively charged glass slides (Thermo Fisher Scientific, Germany) and fixed in acetone for 30 min at − 20 °C. Next, the sections were hydrated for 10 min with PBS, washed with PBST (PBS with 0.05% Tween20) and blocked with 1% BSA (bovine serum albumin) in PBST (blocking buffer) for 1 h. For biotin detection, the sections were incubated with avidin-FITC conjugate (A821, Thermo Fisher Scientific, Germany) diluted in blocking buffer at a final concentration of 2 μg/ml for 1 h. After washing five times with PBST, the sections were mounted (Immunoselect Antifading Mounting Medium DAPI, DIANOVA, Germany) and evaluated using a fluorescent microscope (100×, Axio Scope.A1; Carl Zeiss Microscopy, Germany) equipped with an AxioCam MRc camera (Carl Zeiss Microscopy, Germany).

## Supplementary Information

Supplementary Table S1.

Supplementary Table S2.

Supplementary Figures.

## Data Availability

Mass spectrometry raw data from all measured samples and MaxQuant search results have been deposited to the ProteomeXchange Consortium via the PRIDE^53^ partner repository with the data set identifier PXD031167.
