# Supplementary material for: Comparative proteome analysis of the tegument of male and female adult Schistosoma mansoni
Source: Sci Rep. 2022 May 9;12:7569. doi: 10.1038/s41598-022-11645-3 (PMC9085856; doi:10.1038/s41598-022-11645-3)
Supplement: Supplementary file 3 — Supplementary Figures. [file 41598_2022_11645_MOESM3_ESM.pdf]

Supplementary Figures for

Comparative proteome analysis of the tegument of male and female adult *Schistosoma mansoni*

Franziska Winkelmann[1], Manuela Gesell Salazar[2], Christian Hentschker[2], Stephan Michalik[2], Tomáš Macháček[1,3], Christian Scharf[4], Emil C. Reisinger[1], Uwe Völker[2*], Martina Sombetzki[1*]

[1]Division of Tropical Medicine and Infectious Diseases, Center of Internal Medicine II, Rostock University Medical Center, Rostock, Germany; [2]Department of Functional Genomics, Interfaculty Institute for Genetics and Functional Genomics, University Medicine Greifswald, Greifswald, Germany; [3]Department of Parasitology, Faculty of Science, Charles University, Prague, Czechia; [4]Department of Otorhinolaryngology, Head and Neck Surgery, University Medicine Greifswald, Greifswald, Germany

*corresponding authors, e-mail: martina.sombetzki@uni-rostock.de, voelker@uni-greifswald.de

Supplementary Figures

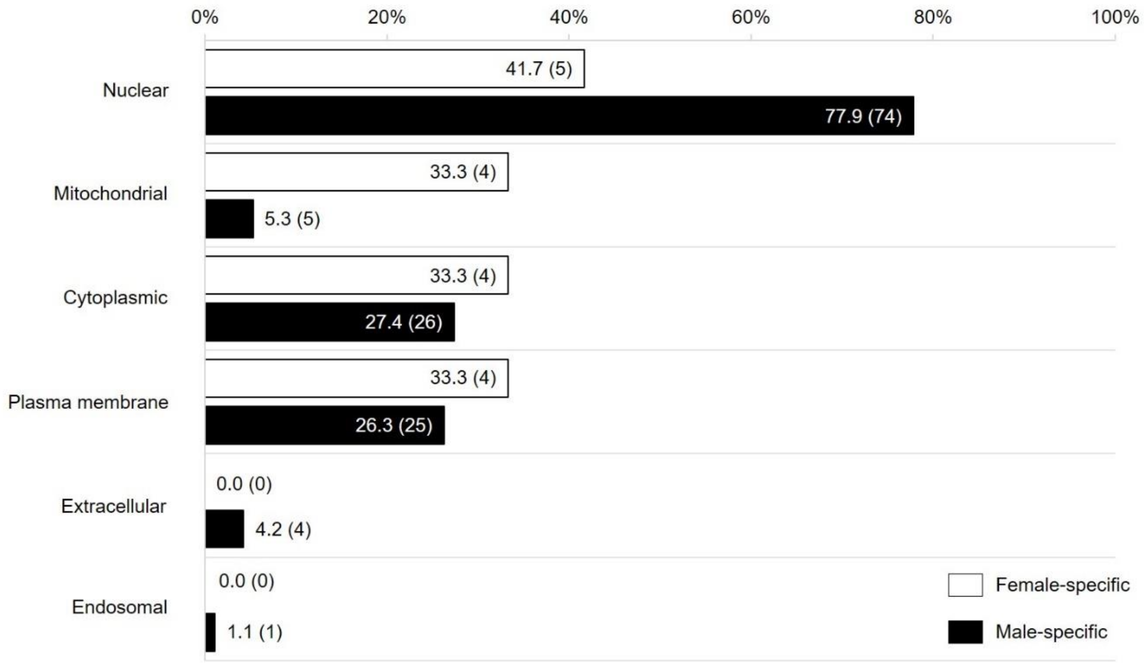

Figure S1. Subcellular localization of biotinylated tegument proteins from male and female *Schistosoma mansoni*. Proteins were analyzed by CELLO2GO and classified into six groups. Number of proteins in brackets.

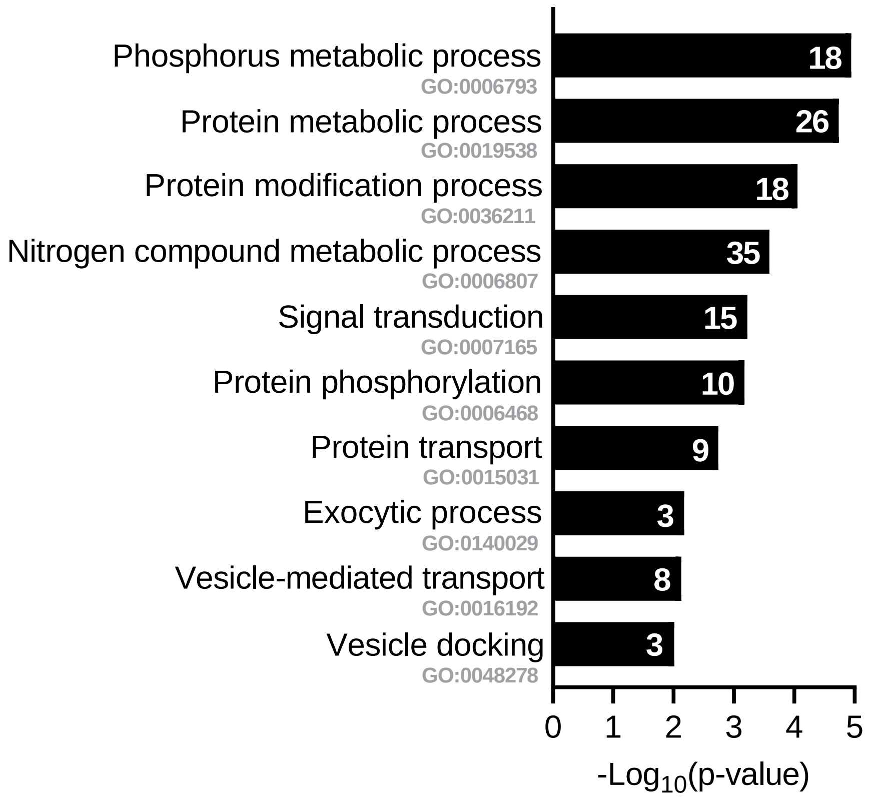

Figure S2. Gene ontology analysis of biotinylated tegument proteins from male and female *Schistosoma mansoni*. Male-specific proteins were assigned to top 10 enriched categories for biological processes. Proteins were analyzed by g:Profiler and plotted usind GraphPad Prism 9. Number of proteins in each category is shown in white.

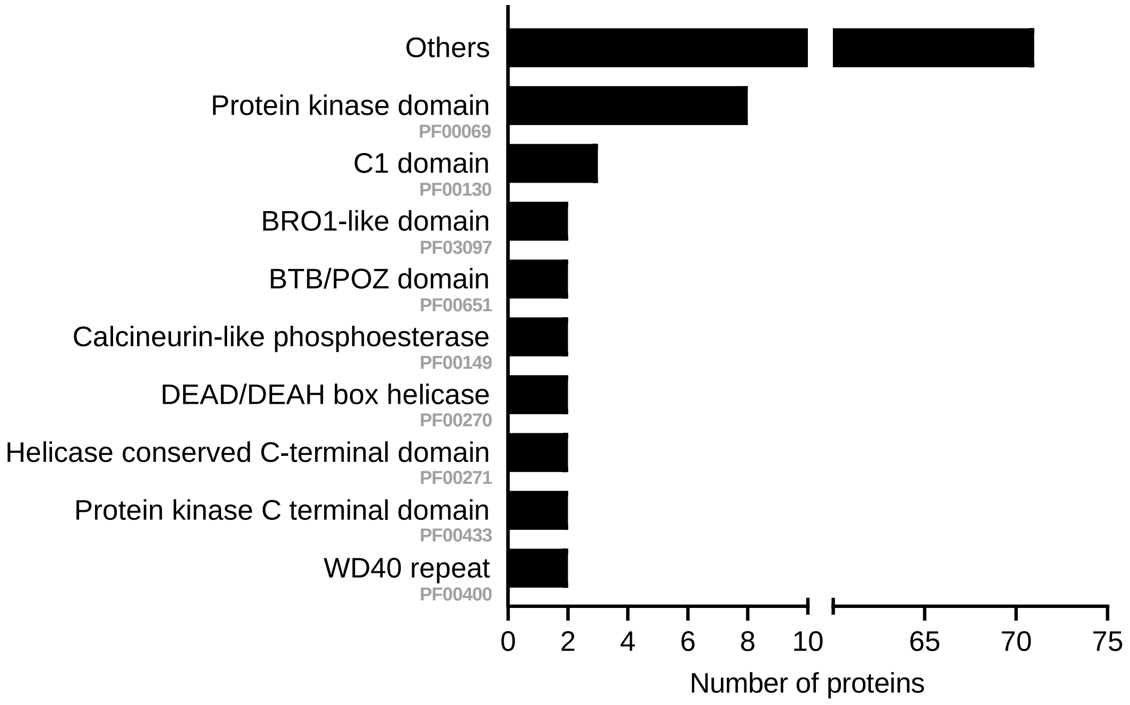

Figure S3. Protein family analysis of biotinylated tegument proteins from male and female *Schistosoma mansoni*. Bar graph summarizing 10 most abundant Protein family domains from the tegument proteins of male-specific adult *Schistosoma mansoni*.
